# P-1174. *In vitro* Activity of Gepotidacin and Comparator Agents Against a Collection of *Escherichia coli* and *Klebsiella pneumoniae* Urine Isolates From the United States During 2023

**DOI:** 10.1093/ofid/ofaf695.1367

**Published:** 2026-01-11

**Authors:** S J Ryan Arends, Renuka Kapoor, Nicole E Scangarella-Oman, Rodrigo E Mendes

**Affiliations:** Element Iowa City (JMI Laboratories), North Liberty, IA; GSK, Atlanta, Georgia; GlaxoSmithKline plc., Collegeville, PA; Element Iowa City (JMI Laboratories), North Liberty, IA

## Abstract

**Background:**

Gepotidacin (GEP) is a recently approved, bactericidal, first-in-class triazaacenaphthylene antibiotic that inhibits bacterial DNA replication by a distinct binding site, unique mechanism of action and provides well-balanced inhibition (for most pathogens) of two different type II topoisomerase enzymes. This study reports on the *in vitro* activity of GEP and other oral antibiotics tested against contemporary *E. coli* (EC) and *K. pneumoniae* (KPN) clinical isolates collected from patients with urinary tract infections (UTI) in the United States (US).

**Figure:**
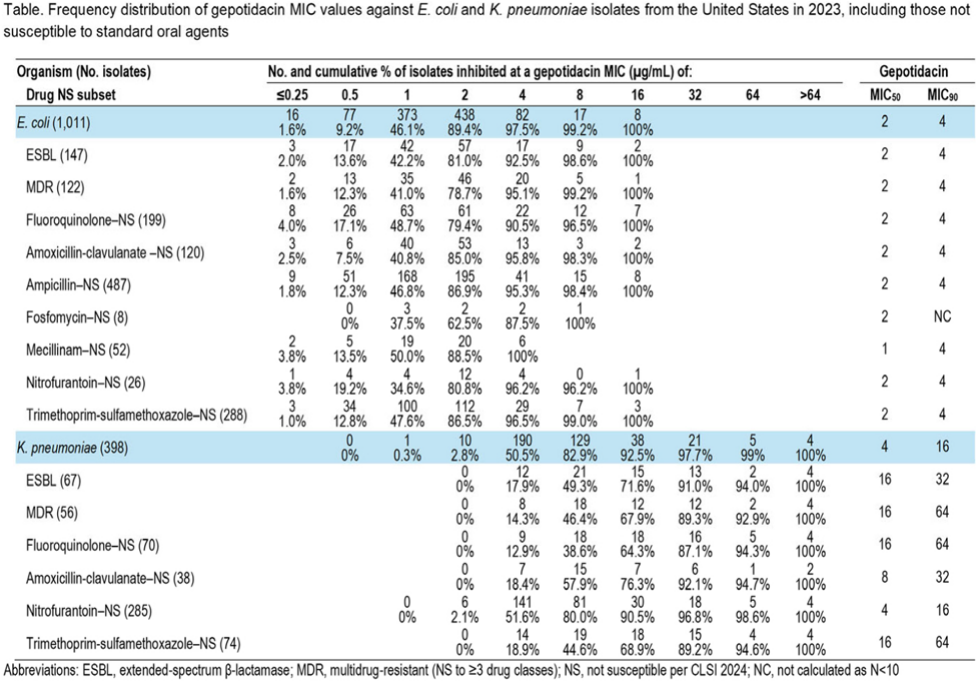

**Methods:**

1,011 EC and 398 KPN isolates were collected in 2023 from 58 medical centers in the US. Isolates were tested for susceptibility by CLSI methods. MIC results for comparator agents were interpreted per CLSI guidelines. Multidrug-resistant (MDR) and extended-spectrum β-lactamase (ESBL) subsets were categorized per CLSI criteria.

**Results:**

GEP (MIC_50/90_, 2/4 µg/mL) displayed activity against EC isolates, with all (100%) isolates having GEP MICs ≤16 µg/mL. Other oral agents demonstrated the following rates of susceptibility: amoxicillin-clavulanate (AMC, 88%), ampicillin (AM, 52%), ciprofloxacin (CIP, 80%), fosfomycin (FOS, 99%), mecillinam (MEC, 95%), nitrofurantoin (FM, 97%), and trimethoprim-sulfamethoxazole (SXT, 72%). GEP maintained similar MIC_50_ values (1–2 µg/mL) and MIC_90_ values (4 µg/mL) against drug not susceptible (NS) subsets of EC. GEP (MIC_50/90_, 4/16 µg/mL) was also active against KPN isolates tested, with 92% of isolates having GEP MICs ≤16 µg/mL. Other oral agents demonstrated the following rates of susceptibility: AMC (90%), CIP (82%), FM (28%), and SXT (81%). The majority of GEP MIC_50/90_ values were within 1- to 2-dilutions (MIC_50/90_ values 4-16/16-64 µg/mL) against drug-NS subsets of KPN.

**Conclusion:**

GEP demonstrated *in vitro* activity against contemporary EC and KPN, including ESBL-producing and MDR isolates. This activity remained unaffected for EC isolates NS to other oral standard-of-care antibiotics and was within 1- to 2- dilutions of the value for the total population for drug-NS subsets of KPN. Susceptibility to other oral agents was ≥80% except for AM and SXT against EC isolates, and was between 80-90% except for FM against KPN isolates.

**Disclosures:**

Renuka Kapoor, PhD, GSK: Employee|GSK: Stocks/Bonds (Public Company) Nicole E. Scangarella-Oman, MS, GSK: Employee|GSK: Stocks/Bonds (Public Company) Rodrigo E. Mendes, PhD, GSK: Grant/Research Support|Shionogi & Co., Ltd.: Grant/Research Support|United States Food and Drug Administration: FDA Contract Number: 75F40123C00140

